# Hyperuricemia beyond gout: an emerging risk factor for sensorineural hearing loss

**DOI:** 10.1007/s00405-026-10426-2

**Published:** 2026-07-19

**Authors:** Gauri Govind, Vijendra S Shenoy, Neehal Zuturu, Vikrant Kamboj, Haneesh Domah, Sanchit Bajpai, Apoorva K V, Navya Parvathareddy, Nayanika Reddy, Ashirwad Sharma

**Affiliations:** 1 Dept of Otorhinolaryngology and Head & Neck surgery, Kasturba Medical College, Mangalore Manipal Academy of Higher Education, Mangalore, Manipal India; 2 Dept of Otorhinolaryngology and Head & Neck surgery, T.S. Misra Medical College, Lucknow, India; 3 Department of Otorhinolaryngology and Head & Neck surgery, Swami Vivekanand hospital and cancer center, Yamunanagar, India; 4 Dept of Otorhinolaryngology and Head & Neck surgery, Ministry of Health and Wellness, Port Louis, Mauritius; 5 Dept of ENT and Head & Neck surgical oncology, Apollo Speciality Hospital, Chennai, India; 6 Dept of Otorhinolaryngology and Head & Neck Surgery, Indira Gandhi Government General Hospital and Post Graduate Institute (IGGGH&PGI), Puducherry, India; 7 Dept of Otorhinolaryngology and Head & Neck Surgery, Apollo Hospitals, Hyderabad, India

**Keywords:** Hyperuricemia, Sensorineural hearing loss, Pure tone audiometry, Distortion product otoacoustic emissions, Cochlear function

## Abstract

**Purpose:**

Hyperuricemia is a common metabolic condition with established systemic effects; however, its potential association with auditory dysfunction has not been clearly defined. This study aimed to evaluate hearing thresholds and cochlear outer hair cell function in individuals with hyperuricemia using subjective and objective audiological assessments.

**Methods:**

A comparative cross-sectional study was conducted involving 130 participants aged 30–55 years, comprising 65 hyperuricemic subjects and 65 age- and gender-matched normouricemic controls. Pure Tone Audiometry (PTA) was performed at frequencies from 250 to 8000 Hz. Distortion Product Otoacoustic Emissions (DPOAE) were recorded at 1000, 2000, 4000, 6000, and 8000 Hz, and signal-to-noise ratio (SNR) values were analyzed. Statistical analysis included Student’s t-test, Mann–Whitney U test, and Chi-square test, with *p* < 0.05 considered statistically significant.

**Results:**

Mean PTA thresholds were higher in hyperuricemic subjects across all tested frequencies compared to controls, with statistically significant differences (*p* < 0.001). Mild sensorineural hearing loss was more frequently observed in the hyperuricemic group. DPOAE analysis demonstrated significantly reduced SNR values, particularly at higher frequencies, and a higher proportion of absent emissions in hyperuricemic subjects. No significant association was observed between hearing loss and age or duration of symptoms.

**Conclusion:**

The findings suggest an association between hyperuricemia and early cochlear dysfunction. Objective measures such as DPOAE may be useful for identifying subclinical auditory involvement in this population.

## Introduction

Hyperuricemia, defined as serum uric acid concentration exceeding 7 mg/dL [1], has emerged as a widespread metabolic disorder with escalating prevalence in developed and developing nations. This increase parallels the global adoption of Western dietary patterns, sedentary lifestyles, and increased alcohol consumption [2]. While the majority of affected individuals remain asymptomatic, the systemic consequences of sustained uric acid elevation extend far beyond articular manifestations, implicating cardiovascular disease, renal dysfunction, metabolic syndrome, and chronic inflammatory conditions [3, 4].

Historically, hearing impairment has not been systematically investigated as a manifestation of hyperuricemia. This oversight likely reflects the limited number of studies addressing this association and the traditional compartmentalization of otologic and rheumatologic pathology. However, emerging evidence suggests that urate accumulation may compromise cochlear integrity through multiple pathophysiological mechanisms [5, 6].

The cochlea occupies a unique anatomical and physiological position within the inner ear, dependent on a single terminal artery—the labyrinthine artery—for its blood supply. This singular vascular supply renders the cochlea acutely sensitive to microcirculatory disturbances. Furthermore, the cochlea sustains the highest metabolic rate of any sensory organ per unit mass, necessitating continuous oxygen and nutrient delivery to maintain the electrochemical gradients that drive mechanotransduction.

Hyperuricemia compromises cochlear microcirculation through multiple mechanisms. Uric acid promotes endothelial dysfunction by inhibiting endothelial cell proliferation and suppressing nitric oxide bioavailability, resulting in reduced vasodilatory capacity [7]. Additionally, Urate crystal deposition activates the NALP3 inflammasome, leading to the release of proinflammatory cytokines such as IL-1β, TNF-α, and IL-6 [8, 9]. Within the cochlea, these cytokines orchestrate a cascade of injury, including leukocyte infiltration, gliosis, and structural tissue degradation.

The outer hair cells (OHCs) of the cochlea, which perform critical roles in frequency selectivity and cochlear amplification through electromotile contractility, are particularly vulnerable to oxidative stress [10]. The prestin protein, which mediates OHC motility, is sensitive to disruption by reactive oxygen species (ROS) generated during hyperuricemic states. Consequently, OHC dysfunction precedes overt sensory cell loss, creating a window of opportunity for therapeutic intervention before permanent structural damage occurs.

Despite the biological plausibility of hyperuricemia-induced cochlear injury, clinical recognition of this association remains limited. The relationship between serum uric acid levels, gout disease, and auditory dysfunction has been addressed in only a handful of studies, primarily case reports or small observational cohorts, leaving the true prevalence and clinical significance of this association poorly characterized. This knowledge gap has important implications for patient management, as identification of a modifiable metabolic risk factor for hearing loss could fundamentally alter the approach to prevention and early intervention in hyperuricemic populations.

The objectives of this study were to: (1) determine whether hyperuricemia functions as an independent risk factor for SNHL through comparative analysis of auditory thresholds between hyperuricemic and normouricemic cohorts; (2) characterize the severity and frequency-specific pattern of hearing impairment; (3) employ objective measures of outer hair cell function (DPOAE) to detect subclinical cochlear dysfunction prior to symptomatic hearing loss manifestation; and (4) assess the association between disease duration and severity of auditory involvement.

## Methods

A comparative cross-sectional study was conducted at Kasturba Medical College Hospital and Government Wenlock Hospital. This study was performed in line with the principles of the Declaration of Helsinki. Approval was granted by the Institutional Ethics Committee of Kasturba Medical College, Mangalore on 16th November 2023.

IEC KMC MLR 11/2023/441. Individuals aged 30–55 years were recruited into two groups: a case group (*n* = 65) with clinical gout or biochemically confirmed hyperuricemia (fasting SUA > 6.8 mg/dL) and an age-matched normouricemic control group (*n* = 65).

To isolate the metabolic effects of hyperuricemia, individuals with potential confounding factors were excluded. Participants with systemic comorbidities such as diabetes mellitus, hypertension, hypercholesterolemia, thyroid disorders, or chronic kidney disease (serum creatinine > 1.2 mg/dL) were excluded. Subjects with otologic factors, including a history of chronic otitis media, prior otologic surgery, head or ear trauma, or use of ototoxic medications, were also excluded. Additionally, individuals with significant occupational noise exposure, defined as exposure exceeding 85 dB for more than 8 h per day for ≥ 1 year, were not included in the study. Furthermore, we excluded participants with a BMI > 25 kg/m², significant smoking or alcohol history, and those using diuretics or aspirin to eliminate lifestyle and pharmacological confounders.

Written informed consent was obtained from all participants prior to enrollment. Each subject underwent an otorhinolaryngological evaluation, including otoscopy and tuning fork tests. Tympanometry was performed to confirm normal middle-ear function (Type A) and exclude conductive pathology. Audiological Assessment Pure tone audiometry (250–8000 Hz) and DPOAE recording (1000–8000 Hz) were performed. To minimize observer bias, examiners were blinded to the participants’ group allocation. Biochemical parameters (SUA, creatinine, urea) were measured from morning venous samples after an 8–12 h fast.

Statistical analysis was performed using Student’s unpaired *t*-test, Mann–Whitney *U* test, and Chi-square test, as appropriate. A p value < 0.05 was considered statistically significant. Given the multiple frequency-specific comparisons in audiometric evaluation, interpretation of results was guided by consistency across clinically relevant high-frequency ranges, which were defined a priori based on known susceptibility patterns. Effect sizes for continuous variables were calculated using Cohen’s d, where values of 0.2, 0.5, and 0.8 indicate small, medium, and large effects, respectively. The 95% Confidence Intervals (CI) for the mean differences were also reported. Data analysis was conducted using IBM SPSS Statistics for Windows, version 29.0 (IBM Corp., Armonk, NY).

## Results

The study included 130 participants with complete audiological assessments, comprising 65 hyperuricemic subjects and 65 normouricemic controls. All analyses were performed on a participant basis, and the findings are presented as the number of participants affected or as mean values, as appropriate.

### Age and symptoms distribution

The mean age of participants in the control and case groups was 45.18 ± 7.96 years and 46.54 ± 7.51 years, respectively, with no statistically significant difference observed between the groups (*p* = 0.307). Serum uric acid concentrations were significantly higher among hyperuricemic subjects (8.11 ± 1.05 mg/dL) compared with normouricemic controls (4.23 ± 0.95 mg/dL; *p* < 0.001). None of the individuals in the control group reported clinical symptoms. Within the case group, 11 participants (16.9%) exhibited symptomatic gout, whereas the majority, 54 subjects (83.1%), were asymptomatic. Despite this, audiological abnormalities were identified in hyperuricemic individuals irrespective of the presence or absence of clinical symptoms.

### Clinical and Biochemical Profile of the Case Group

The case group (*n* = 65) consisted of 47 patients with asymptomatic hyperuricemia, 16 with gout, and 2 with gouty tophi. Most subjects (92.3%, *n* = 60) were not receiving urate-lowering therapy at the time of evaluation. Subgroup analysis revealed no significant differences in mean PTA thresholds between asymptomatic and gouty subjects (*p* = 0.402). While minor variations in DPOAE SNR were noted at 8000 Hz (*p* = 0.033), the overall pattern of cochlear dysfunction remained consistent across the entire hyperuricemic cohort compared to controls.

### PTA vs. hearing loss

**Fig. 1:**
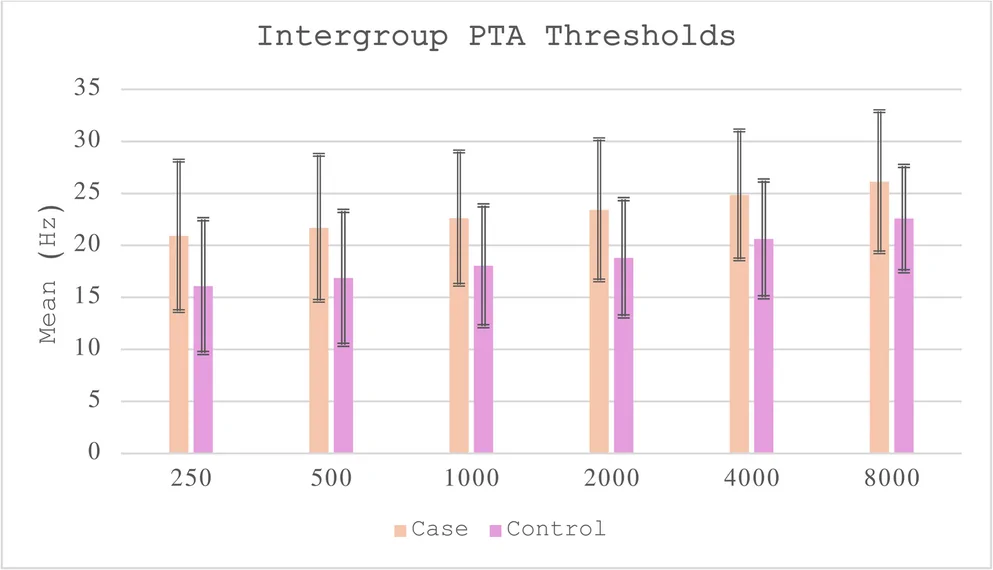
Comparison of mean PTA at various frequencies between Cases and Controls

Pure tone audiometry (250–8000 Hz) demonstrated consistently higher mean hearing thresholds in hyperuricemic cases compared to controls, with all differences being highly significant (*p* < 0.001). Effect size analysis (Cohen’s d: 0.59–0.75) indicated a moderate-to-large clinical impact, and 95% confidence intervals did not cross zero, confirming the reliability of these findings. Based on pure tone averages, mild hearing loss was observed in 41.5% of cases compared to 15.4% of controls, showing a statistically significant difference (*p* = 0.002) (Table 1; Fig. 1).

**Table 1:** Mean pure tone audiometric thresholds (dB HL) across tested frequencies in case and control groups

PTA Frequency	Controls Mean ± SD	Cases Mean ± SD	95% CI	Effect Size (Cohen’s d)	*p*-value
250 Hz	16.08 ± 6.43	20.92 ± 7.23	4.84 (2.46 to 7.22)	0.71	< 0.001
500 Hz	16.88 ± 6.44	21.69 ± 7.02	4.81 (2.47 to 7.15)	0.71	< 0.001
1000 Hz	18.04 ± 5.80	22.62 ± 6.41	4.58 (2.46 to 6.70)	0.75	< 0.001
2000 Hz	18.81 ± 5.63	23.42 ± 6.78	4.61 (2.45 to 6.77)	0.74	< 0.001
4000 Hz	20.62 ± 5.61	24.85 ± 6.20	4.23 (2.18 to 6.28)	0.72	< 0.001
8000 Hz	22.58 ± 5.06	26.12 ± 6.79	3.54 (1.46 to 5.62)	0.59	< 0.001

### Symptom duration in relation to PTA thresholds at 8000 Hz

Participants were evaluated according to the duration of gout-related symptoms and their corresponding average hearing thresholds at 8000 Hz on pure tone audiometry. Patients demonstrating normal hearing at this frequency had a mean symptom duration of 6.05 ± 5.33 months, whereas patients with mild hearing loss showed a longer mean duration of 8.39 ± 8.55 months. However, comparison of mean symptom duration across hearing status categories did not reveal a statistically significant difference (*p* = 0.181).

### Age-related differences in PTA thresholds at 8000 Hz

The relationship between age and hearing status at 8000 Hz, as assessed by pure tone audiometry, was examined in the study population. Patients with normal hearing (based on their average 8000 Hz threshold) demonstrated a mean age of 45.95 ± 7.48 years, whereas patients with mild hearing loss had a similar mean age of 47.32 ± 7.62 years. Statistical analysis showed no significant difference in age between the hearing categories (*p* = 0.469), indicating that age did not significantly influence the severity of hearing loss in this cohort.

### SNR

**Fig. 2:**
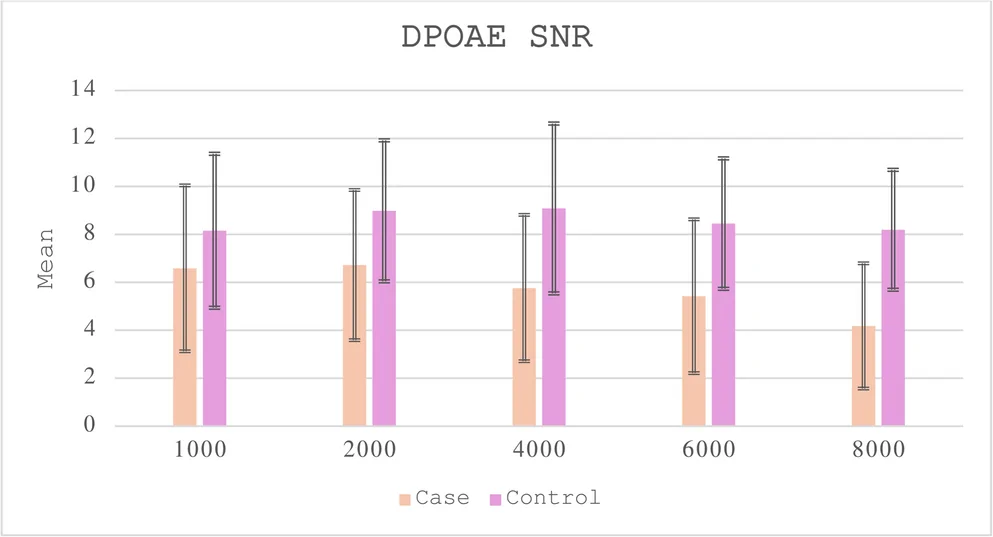
Comparison of mean DPOAE Signal-to-Noise Ratio (SNR) at various frequencies between Cases and Controls

DPOAE-derived signal-to-noise ratio (SNR) values (1000–8000 Hz) were significantly lower in hyperuricemic cases compared to controls (*p* = 0.009 at 1000 Hz; *p* < 0.001 at higher frequencies). The greatest reduction was observed at 8000 Hz (4.18 ± 2.61 dB vs. 8.19 ± 2.50 dB), indicating cochlear dysfunction. Effect sizes increased with frequency, from moderate at 1000 Hz (d = 0.47) to very large at higher frequencies (d = 1.00–1.57), with widening confidence intervals, suggesting greater vulnerability of high-frequency cochlear regions. (Table 2) (Fig. 2).

**Table 2:** Mean signal-to-noise ratio values on distortion product otoacoustic emissions across tested frequencies

Tested SNR Frequency	Controls Mean ± SD	Cases Mean ± SD	95% CI	Effect Size (Cohen’s d)	*p*-value
1000 Hz	8.15 ± 3.21	6.58 ± 3.46	1.57 (0.41 to 2.73)	0.47	0.009
2000 Hz	8.98 ± 2.94	6.72 ± 3.13	2.26 (1.21 to 3.31)	0.74	< 0.001
4000 Hz	9.08 ± 3.54	5.76 ± 3.05	3.32 (2.17 to 4.47)	1.00	< 0.001
6000 Hz	8.45 ± 2.72	5.42 ± 3.21	3.03 (2.00 to 4.06)	1.02	< 0.001
8000 Hz	8.19 ± 2.50	4.18 ± 2.61	4.01 (3.12 to 4.90)	1.57	< 0.001

### DPOAE

**Fig. 3:**
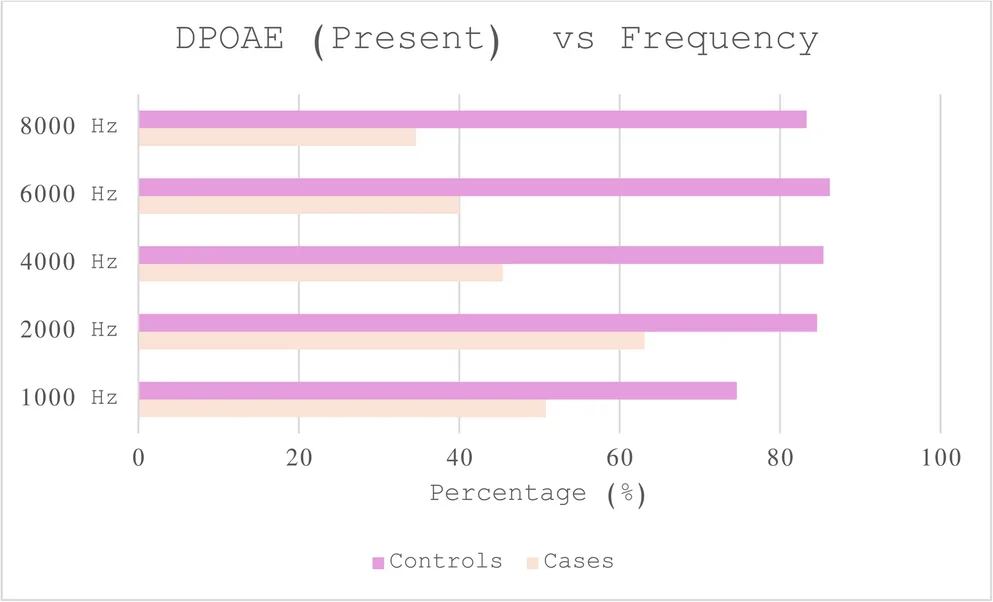
Distribution according to DPOAE at various frequencies between Cases and Controls

DPOAE responses (1000–8000 Hz) were categorized as bilaterally present or absent (in one or both ears). Among the 65 cases, absent responses were observed in 67.7% at 1000 Hz, 55.4% at 2000 Hz, 73.8% at 4000 Hz, and 86.2% at both 6000 and 8000 Hz. Differences between cases and controls were statistically significant across all tested frequencies (*p* ≤ 0.013), reflecting underlying cochlear dysfunction. (Table 3) (Fig. 3).

**Table 3:** Distribution of present and absent DPOAE responses across tested frequencies in case and control groups

Frequency	Result	Cases (*n* = 65) n (%)	Controls (*n* = 65) n (%)	*p*-value
1000 Hz	Bilateral Present	21 (32.3%)	36 (55.4%)	0.013
	Absent (One or both ears)	44 (67.7%)	29 (44.6%)	
2000 Hz	Bilateral Present	29 (44.6%)	47 (72.3%)	0.002
	Absent (One or both ears)	36 (55.4%)	18 (27.7%)	
4000 Hz	Bilateral Present	17 (26.2%)	49 (75.4%)	< 0.001
	Absent (One or both ears)	48 (73.8%)	16 (24.6%)	
6000 Hz	Bilateral Present	9 (13.8%)	48 (73.8%)	< 0.001
	Absent (One or both ears)	56 (86.2%)	17 (26.2%)	
8000 Hz	Bilateral Present	9 (13.8%)	46 (70.8%)	< 0.001
	Absent (One or both ears)	56 (86.2%)	19 (29.2%)	

### Symptom duration in relation to DPOAE outcomes at 8000 Hz

In our study, subjects were analysed based on their duration of symptoms and hearing loss based on DPOAE at 8000 Hz, as most subjects showed low SNR values at this frequency. Patients demonstrating bilaterally present DPOAEs had a mean symptom duration of 10.11 ± 12.15 months, while those with absent emissions (in one or both ears) had a mean duration of 6.57 ± 5.73 months. Comparison of mean duration of symptoms between patients with present and absent DPOAE did not reveal a statistically significant difference (*p* = 0.157).

### Age-related differences in DPOAE responses at 8000 Hz

Participants were further evaluated to determine the relationship between age and DPOAE findings at 8000 Hz, a frequency at which absent responses were most commonly observed. Patients demonstrating bilaterally preserved DPOAE responses had a mean age of 43.22 ± 8.42 years, whereas patients with absent emissions (in one or both ears) had a higher mean age of 47.07 ± 7.30 years. However, statistical comparison of age between the two groups did not show a significant difference (*p* = 0.155), indicating that age alone did not significantly account for the absent DPOAE responses in this cohort.

### Distribution of unilateral and bilateral DPOAE abnormalities

**Fig. 4:**
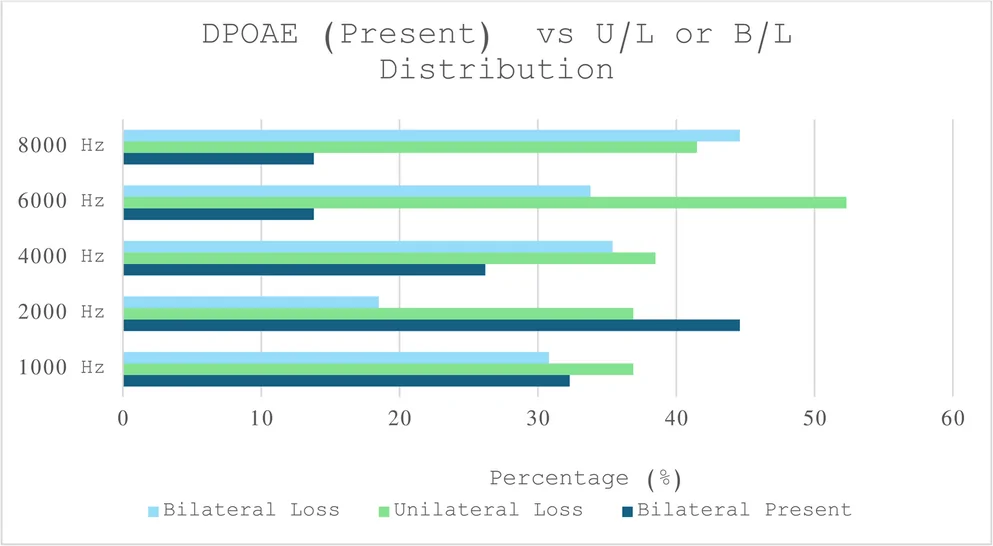
Distribution of Unilateral vs. Bilateral DPOAE Loss at various frequencies among Cases

The laterality of hearing impairment on DPOAE testing was assessed at each frequency and categorized as unilateral or bilateral involvement. At lower frequencies, specifically 1000 Hz and 2000 Hz, unilateral loss was more commonly observed, accounting for 36.9% of cases at both frequencies. In contrast, with increasing frequency, bilateral involvement became more frequent. At 8000 Hz, bilateral DPOAE loss was identified in 29 subjects (44.6%), while unilateral loss was noted in 27 subjects (41.5%). (Table 4) (Fig. 4).

**Table 4:** Frequency-wise distribution of unilateral and bilateral DPOAE loss among case subjects

Frequency	Bilateral Present (Normal) *n* (%)	Unilateral Loss (Absent in 1 Ear) *n* (%)	Bilateral Loss (Absent in 2 Ears) *n* (%)
1000 Hz	21 (32.3%)	24 (36.9%)	20 (30.8%)
2000 Hz	29 (44.6%)	24 (36.9%)	12 (18.5%)
4000 Hz	17 (26.2%)	25 (38.5%)	23 (35.4%)
6000 Hz	9 (13.8%)	34 (52.3%)	22 (33.8%)
8000 Hz	9 (13.8%)	27 (41.5%)	29 (44.6%)

## Discussion

Hyperuricemia is a metabolic disorder characterized by elevated uric acid levels, which can lead to inflammation and oxidative stress. While the articular manifestations of gout are well recognized, the ototoxic potential of this metabolic state is an emerging area of research. The mechanism is thought to involve endothelial dysfunction, oxidative stress, and inflammatory activation within the cochlea. Our study aimed to evaluate the audiological profile of patients with hyperuricemia and gout, utilizing both Pure Tone Audiometry (PTA) and Distortion Product Otoacoustic Emissions (DPOAE) to detect subclinical deficits.

### Distribution of patients and demographic stratification

In the study conducted by Jeong et al. (2023) [6], age was identified as a critical stratification variable, as presbycusis can confound metabolic associations. Our study successfully achieved demographic parity to eliminate this confounder. The mean age in our Case group was 46.54 ± 7.51 years, compared to 45.18 ± 7.96 years in the Control group (*p* = 0.307). This is comparable to the demographic considerations in other studies, such as Sahin et al., though our sample size of 65 cases (130 ears) provides a robust dataset for analysis compared to smaller cohorts in similar literature.

### Age and symptoms distribution

Previous literature suggests that cochlear pathology in metabolic diseases often progresses silently. Abdelkader et al. (2019) [11] noted that high-frequency sensorineural hearing loss often precedes symptom onset in gout patients. Our findings strongly corroborate this. In our study, while only 16.9% of hyperuricemic subjects reported symptoms (e.g., tinnitus, hearing difficulty), objective testing revealed abnormalities in a significantly larger proportion. We observed that age did not significantly correlate with the severity of hearing loss within the case group (*p* = 0.510). This indicates that the observed deficits are metabolic rather than degenerative (presbycusis) in nature, supporting the need for screening even in younger hyperuricemic populations. Subgroup analysis demonstrated that cochlear dysfunction remained consistent across the hyperuricemic cohort regardless of clinical gout symptoms or urate-lowering therapy.

### PTA

According to a study by Shanawar et al. (2024) [12], sensorineural hearing loss was found in approximately 45% of gout patients using PTA. Our study showed a strikingly similar prevalence, with 41.5% of case subjects meeting the criteria for SNHL (elevated PTA in one or both ears). While Sahin et al. (2022) [13] reported threshold elevations primarily at extended high frequencies (> 8000 Hz), our study demonstrated a diffuse elevation of thresholds across the entire frequency spectrum (250–8000 Hz). The mean PTA difference between cases and controls was statistically significant at all tested frequencies (*p* < 0.001). Specifically, at 8000 Hz, we observed a mean threshold of 26.12 dB in cases compared to 22.58 dB in controls. This highlights that while the greatest vulnerability is at high frequencies, the metabolic insult of hyperuricemia affects the cochlea broadly.

### SNR and DPOAE

Reduced Signal-to-Noise Ratio (SNR) in DPOAE is a sensitive marker for outer hair cell dysfunction. Saini et al. (2017) [14] reported significant reductions in DPOAE SNR, predominantly at 5000 and 6000 Hz, in hyperuricemic patients. In our study, significant differences in mean SNR were observed at all frequencies > 1000 Hz (*p* < 0.001). The reduction was most pronounced at 8000 Hz, where the case group showed a mean SNR of 4.18 dB compared to 8.19 dB in controls. Consistent with Süslü et al. [15] (cited in context of Behcet’s/inflammatory disease), who emphasized that low DPOAE responses with normal PTA predict subclinical involvement, we found that 86.2% of our case subjects had absent DPOAEs (in one or both ears) at 8000 Hz. This is notably higher than the percentage of patients showing actual hearing loss on PTA (58.5% had mild loss in at least one ear at 8000 Hz), proving DPOAE is a superior screening tool. We also observed a frequency-dependent laterality pattern: unilateral DPOAE loss was more common at lower frequencies (1000–2000 Hz), whereas bilateral involvement became predominant at 8000 Hz (44.6% bilateral loss). This suggests that metabolic injury may start asymmetrically but progresses to symmetric bilateral damage as the severity or frequency susceptibility increases.

### Limitations

This study utilized convenience sampling from a single tertiary care center, which may introduce selection bias and limit the generalizability of the findings. Additionally, the cross-sectional design allows for the observation of significant associations but precludes establishing a definitive causal relationship between hyperuricemia and cochlear dysfunction. While major comorbidities were excluded, other unmeasured lifestyle factors may influence outcomes. Longitudinal studies are required to confirm if chronic hyperuricemia leads to progressive hearing impairment over time.

### Future Research

Future research on the prevalence of hearing loss in hyperuricemia warrants longitudinal studies with larger sample sizes to determine causality and the potential reversibility of hearing loss with urate-lowering therapy. Integrated biomarker studies measuring inflammatory cytokines and oxidative stress markers would also help elucidate the specific molecular pathways linking uric acid to cochlear injury.

## Conclusion

The analysis and findings of the study demonstrated a strong link between hyperuricemia and sensorineural hearing loss. In summary, we deduced that hyperuricemic patients are significantly more susceptible to cochlear dysfunction compared to age-matched normouricemic controls. This dysfunction is characterized by a diffuse elevation of PTA thresholds and a marked reduction in DPOAE SNRs, particularly at 8000 Hz. DPOAE was found to be a superior diagnostic modality as compared to PTA in screening for subclinical auditory damage. We suggest DPOAE as the preferred screening test to identify early cochlear injury in hyperuricemic patients, enabling timely management and potentially improving quality of life.

## Data Availability

The datasets generated during and/or analysed during the current study are available from the corresponding author on reasonable request.

## Declarations

### Declaration of Large Language Model (LLM) Usage

During the preparation of this work, the author(s) utilized Large Language Models (LLM) solely to improve language and readability. The author(s) reviewed and edited the content as needed and take(s) full responsibility for the content of the publication.
